# Type 2 diabetes increases oocyte mtDNA mutations which are eliminated in the offspring by bottleneck effect

**DOI:** 10.1186/s12958-018-0423-1

**Published:** 2018-11-03

**Authors:** Li Li, Chang-Sheng Wu, Guan-Mei Hou, Ming-Zhe Dong, Zhen-Bo Wang, Yi Hou, Heide Schatten, Gui-Rong Zhang, Qing-Yuan Sun

**Affiliations:** 1 State Key Laboratory of Stem Cell and Reproductive Biology, Institute of Zoology, Chinese Academy of Sciences, Beijing, 100101 China; 2 University of Chinese Academy of Sciences, Beijing, 100101 China; 3 Peking Medriv Academy of Genetics and Reproduction, Beijing, 102629 China; 4 College of Life Science, Qingdao Agricultural University, Qingdao, 266109 China; 5 Department of Veterinary Pathobiology, University of Missouri, Columbia, MO 65211 USA

**Keywords:** Diabetes, Oocyte, mtDNA mutation, Bottleneck

## Abstract

**Background:**

Diabetes induces many complications including reduced fertility and low oocyte quality, but whether it causes increased mtDNA mutations is unknown.

**Methods:**

We generated a T2D mouse model by using high-fat-diet (HFD) and Streptozotocin (STZ) injection. We examined mtDNA mutations in oocytes of diabetic mice by high-throughput sequencing techniques.

**Results:**

T2D mice showed glucose intolerance, insulin resistance, low fecundity compared to the control group. T2D oocytes showed increased mtDNA mutation sites and mutation numbers compared to the control counterparts. mtDNA mutation examination in F1 mice showed that the mitochondrial bottleneck could eliminate mtDNA mutations.

**Conclusions:**

T2D mice have increased mtDNA mutation sites and mtDNA mutation numbers in oocytes compared to the counterparts, while these adverse effects can be eliminated by the bottleneck effect in their offspring. This is the first study using a small number of oocytes to examine mtDNA mutations in diabetic mothers and offspring.

**Electronic supplementary material:**

The online version of this article (10.1186/s12958-018-0423-1) contains supplementary material, which is available to authorized users.

## Background

Diabetes mellitus (DM) has become a worldwide problem. It brings reproductive problems for both men and women. Women with diabetes mellitus often show low quality oocytes, increased chances of miscarriage, and congenital malformations of offspring [1, 2]. Diabetes creates a complex environment, which could cause permanent damage to the oocytes and embryos [3].

Mitochondria are exclusively maternally inherited. All mitochondria in mature oocytes come from primordial germ cells (PGCs), which have only a few hundred mitochondria [4]. During the one-cell zygote development to preimplantation blastocysts, cells divide persistently with no mitochondrial replication. Mitochondria transmission to offspring from only a small proportion of mitochondria in maternal PGCs is referred to as the mitochondrial genetic ‘bottleneck’. During embryo development, continuous cleavage with limited maternal mitochondria produces cells with different mitochondria resulting in organ and tissue heterogeneity. Therefore, there are bottlenecks during both oogenesis and cleavage stages. Studies proposed two mechanisms of mtDNA bottleneck transmissions. One is unequal partitioning of mtDNA during cell division and the other is selection of a small subpopulation of mitochondria. This could produce rapid genotypic shifts within a single generation [5].

Mitochondrial DNA (mtDNA) has a circular double stranded configuration comprising approximately 16,569 base pairs in rodent mature oocytes; mtDNA copy number must be 40,000–50,000 in order to sustain embryo development [6]. Since it has no repair system, mtDNA is easily mutated and its mutation rate might be 5–10 fold of that of nuclear DNA (nDNA) [7]. Generally, mtDNA in cells is homogeneous, but when mtDNA mutation occurs it becomes heterogeneous. mtDNA has thousands of copies in a cell, and coexistence of a few mtDNA mutations with wild type mtDNA does not cause abnormality, unless the mutation ratio reaches a threshold usually as high as 60–90% [8, 9], with some exceptions [10].

Many studies have proved that in both normal human cells and tumor cells, mtDNA mutations exist [11, 12]. The mtDNA bottleneck exerts a strong purifying selection in the maternal germ line to avoid mtDNA mutation transmissions to offspring [13, 14]. Diabetes affects oocyte quality and fertility by affecting mitochondrial distribution and activity, but whether it causes mtDNA mutations, and if it does, whether the mtDNA mutations are transmitted to offspring, are not known. In this study, we generated a diabetes mouse model to explore mtDNA mutations in oocytes in diabetic mice and their offspring. We showed that mtDNA mutations increased in the T2D mouse model, but the mitochondrial bottleneck could bring clearance of mtDNA mutations in the offspring.

## Methods

All C57BL/6 mice were bought from SPF (Beijing) Biotechnology Co., Ltd. and maintained at 12D:12 L in a temperature-controlled room. All procedures described were reviewed and approved by the ethical committee of the Institute of Zoology, Chinese Academy of Sciences. All animal care and use procedures were in accordance with guidelines of the Institutional Animal Care and Use Committee of the Institute of Zoology, Chinese Academy of Sciences.

### Diabetic mouse model

We generated a type 2 diabetes (T2D) mouse model according to previous studies, with slight changes [15, 16]. Female F0 mice were placed into cages with HFD (high fat diet, D12492, 60% kcal fat) or control diet from 3 weeks old until 20 weeks of age. At 16 weeks mice fed with HFD were injected intraperitoneally with a sub-diabetogenic dose of STZ (100 mg/kg body weight) and kept on the same diet until 20 weeks. We examined fasting blood glucose (overnight fasting), and only glucose levels at ≥17 mM were considered as diabetes. The body weight was checked at 12 weeks\16 weeks\20 weeks.

### Generation of offspring

To generate F1 mice, type 2 diabetes mice were mated with normal male C57BL/6 mice at 20 weeks. On the next morning, if a vaginal plug was observed in female mice, mice were recognized as pregnant. Female ICR mice were mated with normal male ICR mice as foster nursing mothers.

### Glucose tolerance test and insulin resistant test

In type 2 diabetes mice and their offspring, glucose tolerance test (GTT) and insulin resistant test (ITT) were conducted. For GTT and ITT, mice were fasted for 12 h or 2 h, respectively, and 2 g/kg glucose or 1 IU/kg insulin was injected intraperitoneally. Blood glucose from tail blood was monitored at 0 min, 15 min, 30 min, 45 min, 60 min, 90 min and 120 min, respectively, by a glucometer (Accu-CHEK active; Roche Diagnostic).

### Oocyte collection

To collect MII oocytes, mice were superovulated by intraperitoneal injection of 8 IU equine chorionic gonadotropin (PMSG; Tianjin Animal Hormone Factory); 46–48 h later 8 IU human chorionic gonadotropin (hCG; Tianjin Animal Hormone Factory) was injected intraperitoneally. The oocytes were collected 13–14 h after hCG injection.

### mtDNA mutation detection

For mtDNA mutation detection, a total of 12–15 oocytes were used in each group. DNA amplification was carried out through whole genome amplification kit (Peking Jabrehoo Med Tech Co.,Ltd), and library preparation was performed by Easy Prep PGS DNA Lib Prep Kit for Illumina (Peking Jabrehoo Med Tech Co., Ltd). The wild-type mouse mtDNA sequence index was built by bowtie2 software [17], and compared to our sequencing data. Then variation files were obtained through samtools [18, 19], which is annotated by VEP (variant Effect Predictor) [20] to achieve the number of mutations.

### Statistical analysis

Data from repeated experiments were analyzed by independent sample T test with SPSS software. Differences at the *P* <0.05 level were considered statistically significant.

## Results

### Establishment of the diabetes mouse model

**Fig. 1:**
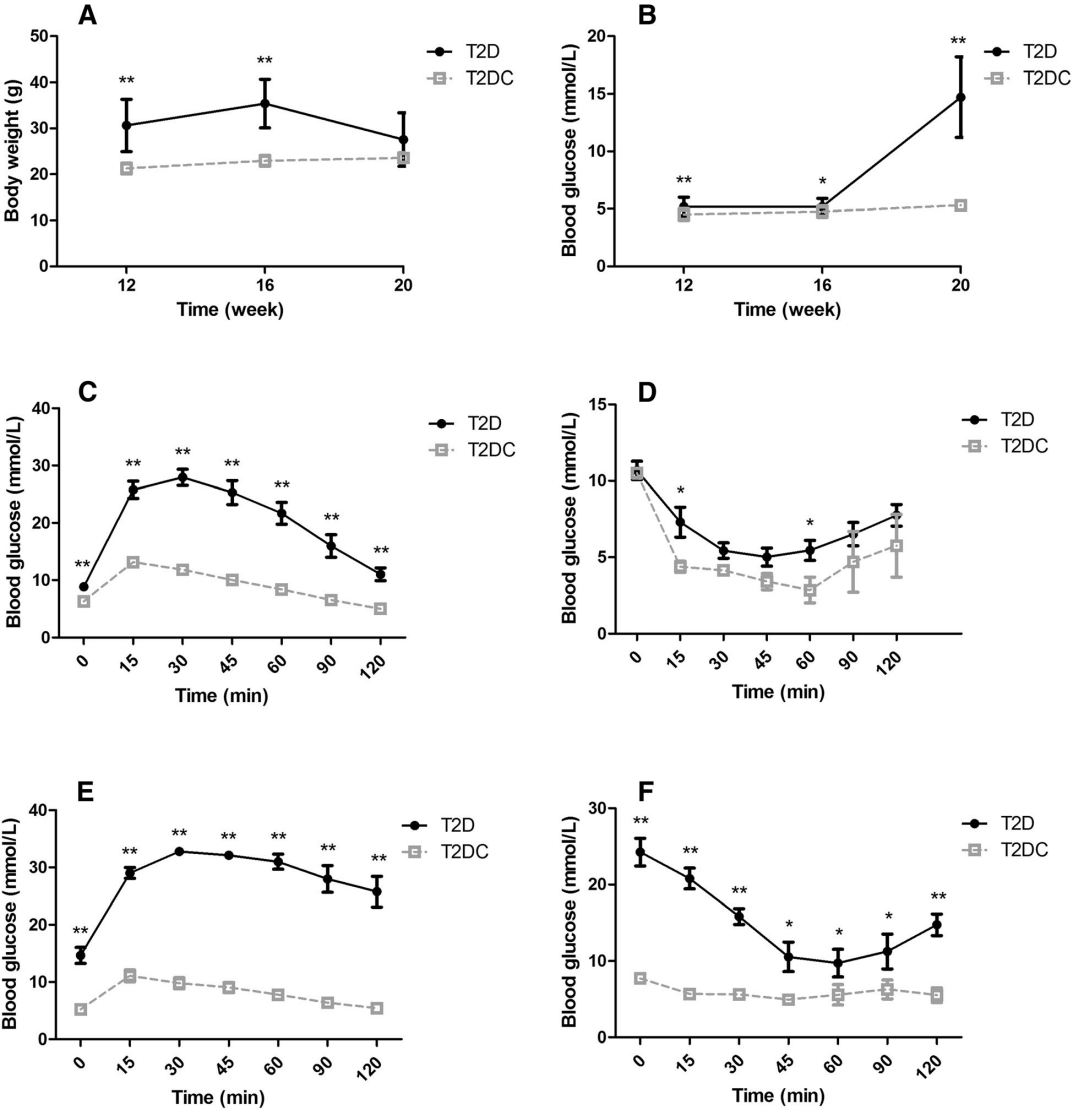
A sketch of the T2D mouse model**.** (**a**) Body weight and (**b**) blood glucose were significantly increased (P<0.05) in T2D mice compared to the T2 DC group. (**c**) Glucose tolerance test (GTT) and (**d**) Insulin resistance test (ITT) at 16 weeks in T2D and T2 DC groups. T2D mice showed glucose intolerance and insulin resistance. After injection of STZ, at 20 weeks, glucose tolerance test (GTT) (**e**) and insulin resistance test (ITT) (**f**) are also seen in T2D mice. T2D mice showed persistent glucose intolerance and insulin resistance

To explore the effect of diabetes on oocyte mtDNA mutations, we generated a T2D mouse model. T2D mouse model was obtained by feeding a HFD combined with STZ injection at a low dose. The body weight of T2D mice was significantly higher than that of the T2D control (T2 DC), reaching a peak at 16 weeks (Fig. 1a). After injection of STZ, the body weight of T2D mice began to decrease, suggesting that diabetes mellitus is a wasting disease. The fasting blood glucose in T2D mice was significantly higher than that of T2 DC mice in all studies, especially after injection of STZ, reaching a 2.7 fold increase (T2D: 14.71 ± 3.52;T2 DC:5.3 ± 0.31) (Fig. 1b).

At 16 weeks, we tested GTT and ITT between T2D and T2 DC mice. T2D mice showed glucose intolerance (Fig. 1c) and insulin resistance (Fig. 1d). After injection of STZ, at 20 weeks, impaired glucose metabolism (Fig. 1e) and insulin resistance (Fig. 1f) were also observed inT2D mice.

### Diabetes mice have a low fecundity

**Fig. 2:**
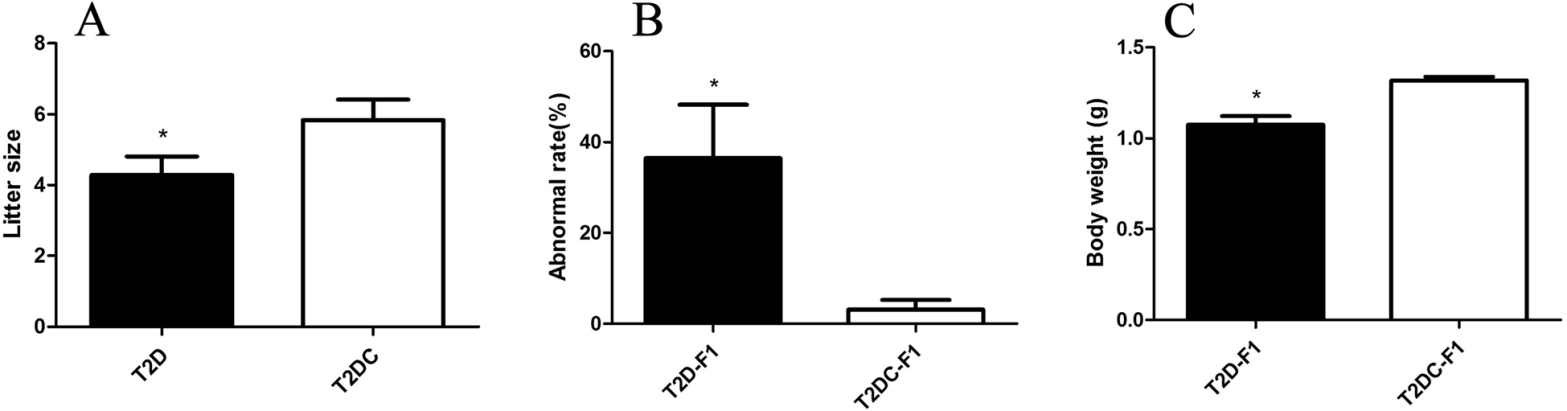
T2D mouse model has a low fecundity. (**a**) The litter size was significantly decreased in T2D mice compared to the T2 DC group (*P*<0.05). (**b**) T2D-F1 newborn mice had a significantly higher malformation rate and significantly decreased low (**c**) birth weight compared to the T2 DC group (*P*<0.05)

At 20 weeks, T2D and T2 DC were mated with normal male C57BL/6 mice. T2D mice did mate with male mice, as indicated by the appearance of a vaginal plug, but they had a higher pseudopregnancy rate compared to T2 DC mice (pregnancy rate: T2D 15.79%; T2 DC 44.68%) (Table 1). In other words, fewer T2D mice with vaginal plugs became pregnant. In the parturation time T2D mice showed dystocia, and we could not obtain F1 newborns. So we used cesarean section to obtain F1 newborns, and ICR primipara that had given birth to newborns 1–2 days earlier were used as foster nursing mothers. To validate the results, F1 newborns from T2 DC mice were also obtained by cesarean section and foster nursing. When T2D and T2 DC mice were processed for cesarean section to obtain F1 newborns, T2D mice had a significantly lower litter size compared to T2 DC mice (Fig. 2a). And the F1 newborn mice had a significantly higher malformation rate (Fig. 2b) and significantly lower birth weight (Fig. 2c).

**Table 1:** Pregnancy rate in T2D mice

	No. Mated	No. pregnancy	Pregnancy rate (%)
T2D	57	9	15.79
T2 DC	47	21	44.68

### Offspring of diabetes mice grow normally

**Fig. 3:**
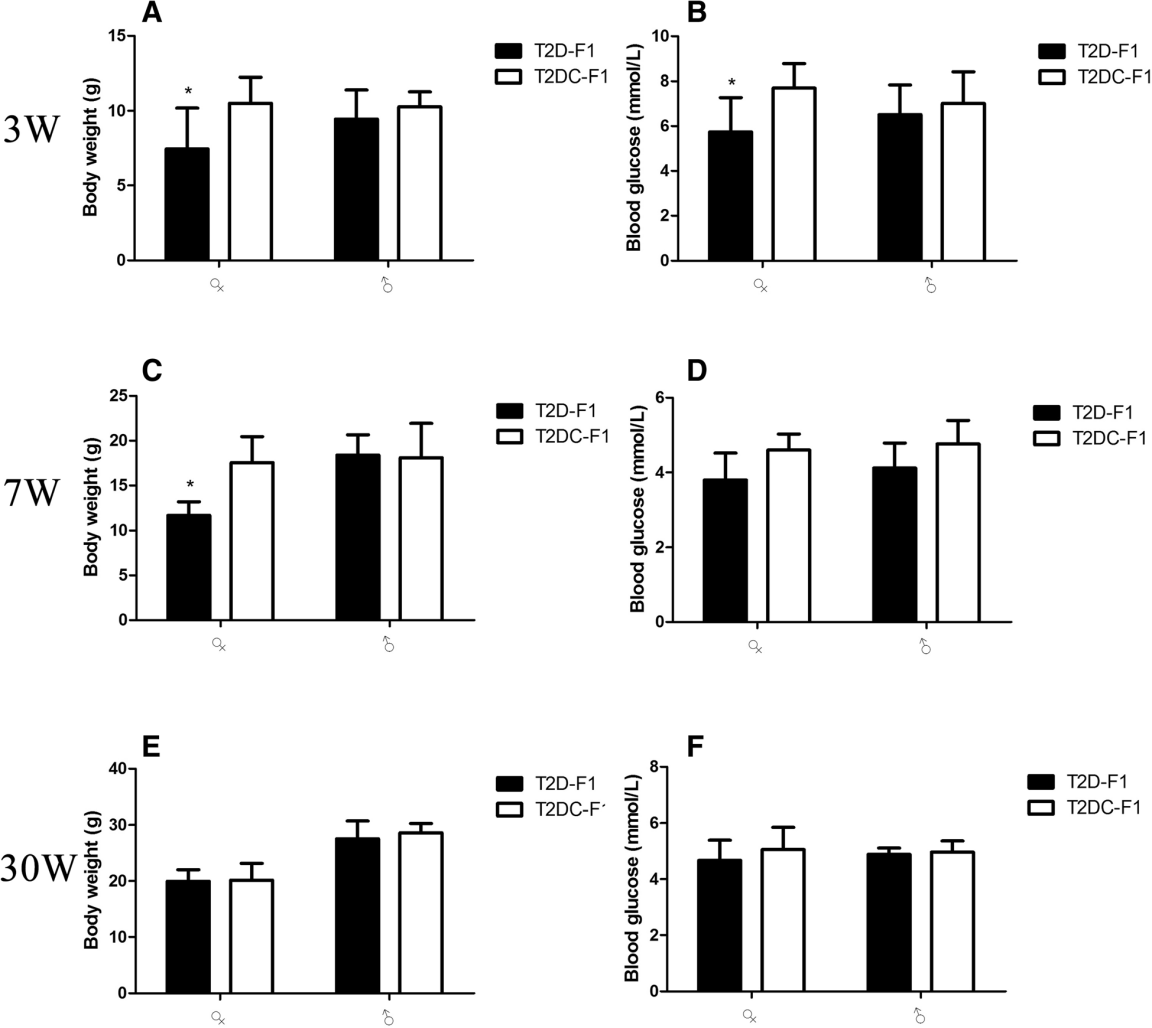
T2D-F1 body weight and blood glucose**.** Female T2D-F1 group had significantly lower (**a**) body weight and (**b**) blood glucose (*P*<0.05) at weaning. At 7 weeks, female T2D-F1 mice showed significantly lower (**c**) body weight compared to female T2 DC-F1 mice. (**d**) Blood glucose in the two groups showed no difference in male\female mice. At 30 weeks, there were no differences in body weight (**e**), and blood glucose (**f**) between the two groups

Both T2D-F1 and T2 DC-F1 mice were foster-nursed by ICR mice. At 21 days, all F1 mice were weaned. Body weight and blood glucose were checked in both male and female F1 mice. The female T2D-F1 group had significantly lower body weight and blood glucose. The body weight and blood glucose in male F1 mice showed no significant difference (Fig. 3a, b).

**Fig. 4:**
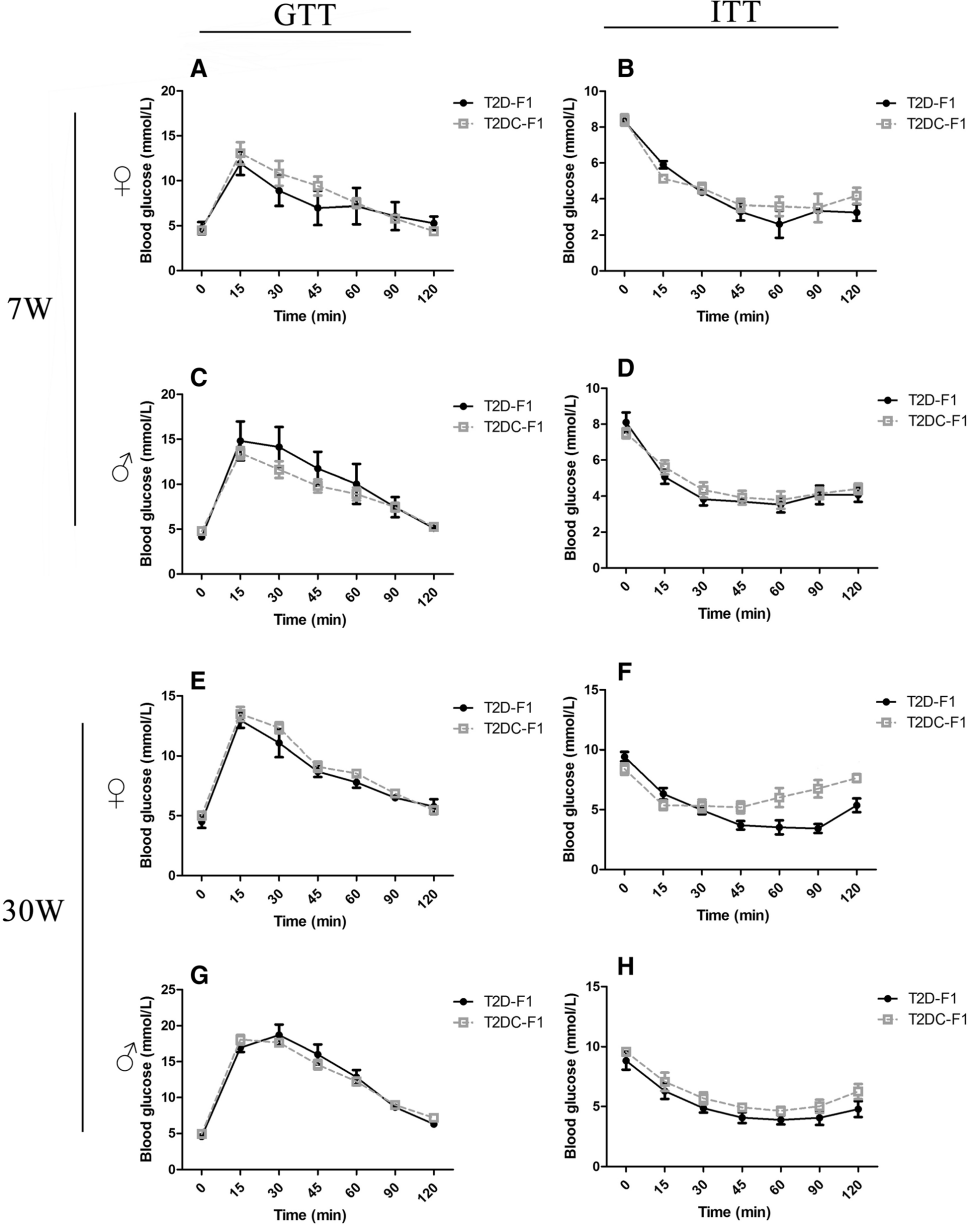
GTT and ITT in growing F1 mice**.** (**a**) Female GTT and (**b**) ITT at 7 weeks, male (**c**) GTT and (**d**) ITT at 7 weeks. (**e**) Female GTT and (**f**) ITT at 30 weeks, male (**g**) GTT and (**h**) ITT at 30 weeks. At 7 or 30 weeks, female or male T2D-F1 mice showed no difference in glucose tolerance test and insulin resistance test

At 7 weeks, F1 mice were sexually mature, and their body weight, blood glucose, GTT and ITT were tested. In female T2D-F1 mice, there was a significantly decreased body weight compared to T2 DC-F1 female mice (Fig. 3c). There was no significant difference in blood glucose (Fig. 3d), GTT and ITT between the two groups. In male F1 mice, there was also no significant difference in body weight (Fig. 3c), blood glucose (Fig. 3d), GTT and ITT between the two groups (Fig. 4a-4d).

At 30 weeks, F1 mice entered the post-adolescence stage of life, and body weight, blood glucose, GTT and ITT were tested. There was no significant difference in body weight (Fig. 3e), blood glucose (Fig. 3f), GTT and ITT in female and male F1 mice between the two groups (Fig. 4e-4h).

### mtDNA mutation in oocytes

**Fig. 5:**
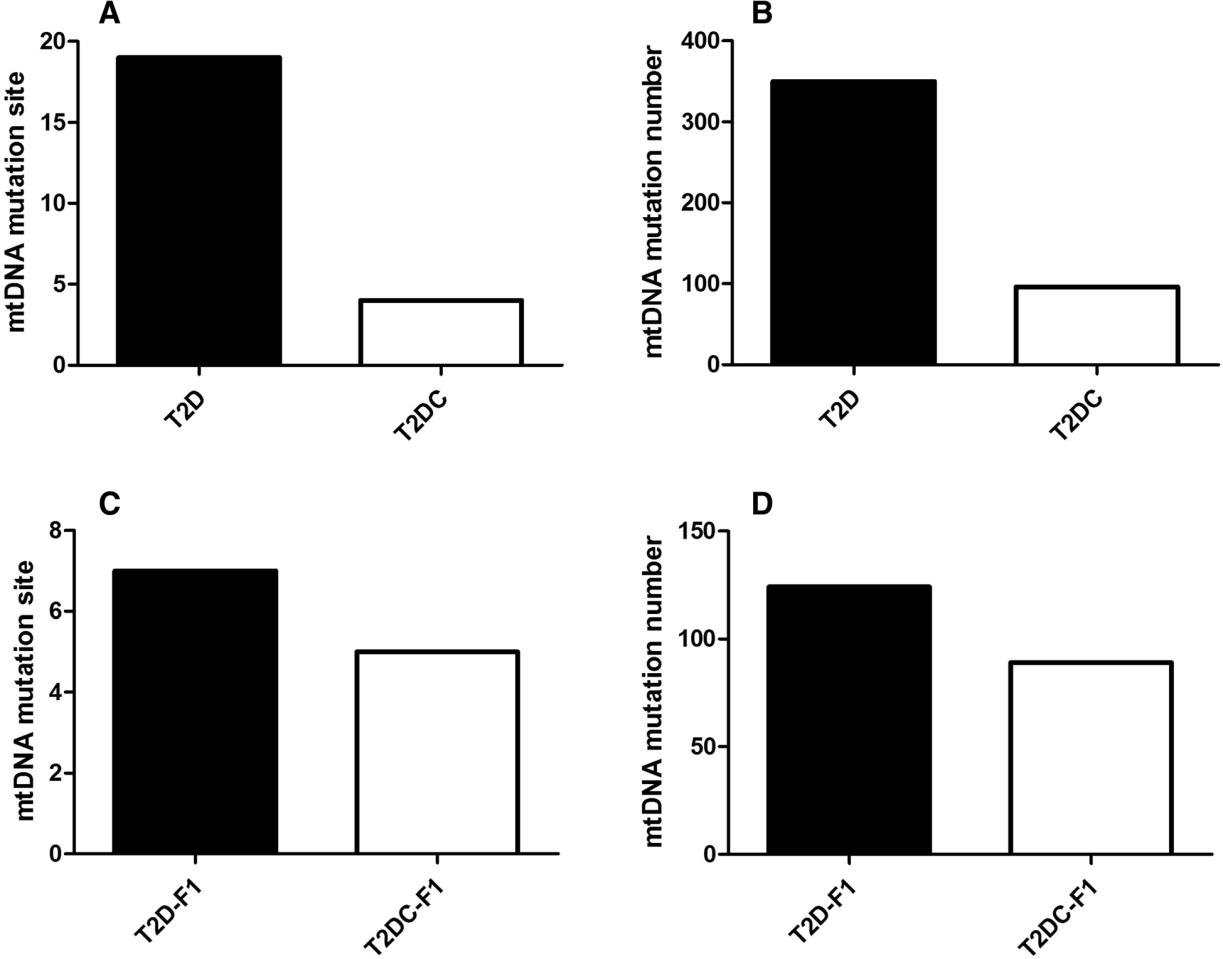
mtDNA mutations in oocytes. (**a**) mtDNA mutation sites and (**b**) mtDNA mutation numbers were increased in the T2D group compared to the T2 DC group. (**c**) mtDNA mutation sites and (**d**) mtDNA mutation numbers in the T2D-F1 group and the T2 DC-F1 group

For T2D and T2 DC groups, we obtained MII oocytes at 20 weeks, which were used for analysis of mtDNA mutations. We found that both mtDNA mutation sites and mtDNA mutation numbers were increased in the T2D group compared to the T2 DC group (Fig. 5a, b; Additional file 1).

For T2D-F1 and T2 DC-F1 mice, the MII oocytes were collected at 30 weeks for mtDNA mutations analysis. The T2D-F1 oocytes had fewer mtDNA mutation sites and mtDNA mutation numbers when compared to T2D oocytes, suggesting the elimination of mutated mitochondria. The T2 DC oocytes and T2 DC-F1 oocytes showed similar mtDNA mutations (Fig. 5c, d; Additional file 1).

## Discussion

Diabetes mellitus is a chronic endocrine metabolic diseases, which can cause many complications, such as diabetic eye disease, diabetic nephropathy and diabetic foot disease [21, 22]. Many studies suggested that diabetes caused reproduction ability decline, both for males and females [23–27], and it also induced diabetic embryopathy [1, 28]. Diabetes can foster a eutrophy environment, high glucose, hyperlipidemia, which impair mitochondrial function. Whether diabetes causes mitochondrial mtDNA mutations is not known. We generated a T2D mouse model, and showed that, in addition to glucose intolerance, insulin resistance, and low fecundity, T2D also increased mtDNA mutation sites and mutation numbers. The mitochondrial bottleneck, existing in oogenesis and embryo cleavage periods for clearance of mtDNA mutations [13, 14], eliminated mtDNA mutations in T2D-F1 mice.

Offspring of T2D diabetic individuals have a high rate of glucose intolerance, insulin resistance, and obesity. For T2D-F1 mice, a lower birth weight was seen compared to the T2 DC-F1 group. At 3 weeks female T2D-F1 mice also showed a lower body weight and blood glucose level. When the two groups were further fed at the same nutrition level, the body weight of T2D-F1 showed a rapid growth phenotype. This might be related to catch-up growth and protective effects of estrogen [29, 30]. Diabetes often occurs at an older age. In our study, offspring of T2D did not develop obesity or high blood glucose, and this may be because they were in a post-adolescence stage of life, not in an advanced stage.

In 1988, it was found for the first time that mtDNA mutations could cause human diseases [31, 32]. In humans, 1 in 400 individuals within populations could inherit maternal mtDNA mutations [33], and 1 in 4300 individuals within populations might have mtDNA diseases [34, 35]. Studies in humans suggested that not only in patients and aging individuals mtDNA mutations exist, but normal healthy individuals also harbor mtDNA mutations [12]. However, individuals with mtDNA mutations usually display normal phenotypes. When a threshold exists mtDNA mutations may induce diseases. Studies showed that an mtDNA deleterious threshold was 60%, and that of point mutations was 90%. But a clinical case suggested that mtDNA mutations <25% had affected tissues [10]. In our study, we detected increased mtDNA mutations in oocytes of T2D mice, suggesting that diabetes might induce mtDNA mutations in reproductive cells.

Mitochondria are maternally inherited. In theory, maternal mtDNA mutations may also be transmitted to the offspring. But mtDNA diseases occur in only 1 in 4300 individuals within human populations. This is owing to maternal mtDNA mutation clearance and in vivo mtDNA mutation threshold effects. mtDNA mutation threshold was usually 60–90%. In view of this fact, it is hard to reach mtDNA mutation threshold in vivo. There are several mechanisms to eliminate mtDNA mutations [36–38]. Mitochondria may be in an active state of fusion, biogenesis and mitophagy, which could eliminate mtDNA mutations. In vivo, mtDNA mutations could be recovered via recombination-mediated repair which may only exist in plants, and in many cases mtDNA mutation elimination is achieved through autophagy and bottleneck-mediated selection [39]. Our study showed that T2D-F1 oocytes displayed fewer mtDNA mutation sites and mtDNA mutation numbers compared to the maternal oocytes. T2D-F1 mice showed almost the same mtDNA mutation level as that seen in the control, which means that bottleneck mediation might eliminate mtDNA mutations in T2D offspring.

## Conclusion

In conclusion, oocytes from T2D mice have increased mtDNA mutation sites and mtDNA mutation numbers compared to the counterparts, while these adverse effects can be eliminated by the bottleneck effect in their offspring. This is the first study using a small number of oocytes to examine mtDNA mutations in diabetic mice.

## Additional file

Additional file 1: Supplement data. (XLSX 12 kb)
